# 

**Published:** 1952-07

**Authors:** 


### Contents

| | | Page |
|---|---|---|
| EDITORIAL | | 75 |
| THE DIAGNOSIS OF ABDOMINAL EMERGENCIES IN CHILDREN | *Mr. R. E. Horton* | 77 |
| UNEXPLAINED INCREASE OF FATAL PULMONARY EMBOLUS AT THE BRISTOL ROYAL INFIRMARY IN 1950 | *Mr. Michael G. Wilson* | 82 |
| GARGOYLISM | *Dr. C. R. Croft* | 90 |
| THE RELATION BETWEEN HYPERTENSION AND THE KIDNEY | *Dr. Harry Sosnowick* | 93 |
| THE SURGICAL TREATMENT OF BRONCHIECTASIS | *Mr. J. L. Griffith* | 98 |
| UNILATERAL PAPILLARY NECROSIS OF KIDNEY IN A DIABETIC | | 100 |
| VISIT OF DR. ALFRED BLALOCK | | 102 |
| REPORTS OF MEETINGS | | 104 |
| DEVON AND EXETER MEDICO-CHIRURGICAL SOCIETY | | |
| SURGICAL CLUB OF SOUTH WEST ENGLAND | | |
| SOUTH WEST ORTHOPAEDIC CLUB | | |
| SOUTH WEST REGIONAL PAEDIATRIC CLUB | | |
| SYMPOSIUM ON THE SUPRARENAL CORTEX | | |
| ANNOTATION | | 109 |
| THE TEACHING OF ANAESTHESIA IN EUROPE | | |
| CORRESPONDENCE | | 110 |
| MATERNITY SERVICE IN BRISTOL | Prof. G. G. Lennon | |
| APPOINTMENTS | | 112 |
| NOTES AND NEWS | | 114 |
| SENIOR DEGREES AND DIPLOMAS | | |
| PRIZES | | |
| UNIVERSITY OF BRISTOL | | |
| BRISTOL MEDICO-CHIRURGICAL SOCIETY | | |
| WESSEX RAHERE CLUB | | |

